# Vertical Distribution of Microbial Eukaryotes From Surface to the Hadal Zone of the Mariana Trench

**DOI:** 10.3389/fmicb.2018.02023

**Published:** 2018-08-28

**Authors:** Zhimeng Xu, Min Wang, Wenxue Wu, Yifan Li, Qian Liu, Yuye Han, Yong Jiang, Hongbing Shao, Andrew McMinn, Hongbin Liu

**Affiliations:** ^1^College of Marine Life Sciences, Ocean University of China, Qingdao, China; ^2^Division of Life Science, The Hong Kong University of Science and Technology, Kowloon, Hong Kong; ^3^Institute of Evolution and Marine Biodiversity, Ocean University of China, Qingdao, China; ^4^Institute for Geophysics and Meteorology, University of Cologne, Cologne, Germany; ^5^Institute for Marine and Antarctic Studies, University of Tasmania, Hobart, TAS, Australia

**Keywords:** microbial eukaryotes, size fraction, diversity, community structure, hadal zone, biotic associations, Mariana Trench

## Abstract

Marine microbial eukaryotes are ubiquitous, comprised of phylogenetically diverse groups and play key roles in microbial food webs and global biogeochemical cycling. However, their vertical distribution in the deep sea has received little attention. In this study, we investigated the composition and diversity of the eukaryotes of both 0.2–3 μm and >3 μm size fractions from the surface to the hadal zone (8727 m) of the Mariana Trench using Illumina MiSeq sequencing for the 18S rDNA. The microbial eukaryotic community structure differed substantially across size fractions and depths. Operational taxonomic unit (OTU) richness in the >3 μm fraction was higher than that in the 0.2–3 μm fraction at the same depth. For the 0.2–3 μm fraction, sequences of Retaria (Rhizaria) were most abundant in the surface water (53.5%). Chrysophyceae (Stramenopiles) sequences dominated mostly in the samples from water depths below 1795 m. For the >3 μm fraction, sequences of Dinophyceae (Alveolata) were most abundant in surface waters (49.3%) and remained a significant proportion of total sequences at greater depths (9.8%, on average). Retaria sequences were abundant in samples of depths ≥1000 m. Amoebozoa and Apusozoa sequences were enriched in the hadal sample, comprising 38 and 20.4% of total sequences, respectively. Fungi (Opisthokonta) sequences were most abundant at 1759 m in both size fractions. Strong positive associations were found between Syndiniales (mainly MALV-I and MALV-II) and Retaria while negative associations were shown between MALV-II and Fungi in a co-occurrence analysis. This study compared the community structure of microbial eukaryotes in different zones in the deep sea and identified a distinct hadal community in the larger size fraction, suggesting the uniqueness of the eukaryotes in the biosphere in the Mariana Trench.

## Introduction

Microbial eukaryotes play a fundamental role in marine ecosystems by supporting global biological and geochemical processes, especially in microbial food webs (Worden et al., 2015). Microbial eukaryote diversity is high and much of this has been discovered using molecular approaches such as high throughput sequencing (HTS). Picoeukaryotes (0.2–3 μm) have been widely reported as being abundant throughout the water column, playing key roles in primary production and mineral cycling (Massana, 2011). However, most picoeukaryotes are relatively new to science and little is understood of their ecology (Worden et al., 2004). Because there are far fewer 18S rDNA copies in picoeukaryotes compared with larger eukaryotes (Zhu et al., 2005), the estimation of microbial eukaryotic community structure may be skewed in those studies using DNA sequencing. Thus, characterizing diversity and community structure of microeukaryotes by size fractionation is a necessary step toward quantifying the ecological importance of these microeukaryotes and studying the interactions between them (Parris et al., 2014).

Deep-sea (>200 m) environments cover more than 65% of the earth’s surface and more than 95% of the global biosphere (Herring, 2001; Corinaldesi, 2015). Depth plays a key role in shaping the diversity and community structure of microbial eukaryotes, and some groups of microbial eukaryotes have apparent depth-related distributions (Countway et al., 2010; Schnetzer et al., 2011). Previous studies have also revealed a variety of eukaryotic community structures in extreme habitats (e.g., Edgcomb V. et al., 2011; Amaral-Zettler, 2013; Logares et al., 2014; Parris et al., 2014), and shown that deep-sea microbial eukaryotic communities are significantly different from those in surface waters. For instance, several groups, such as Rhizaria and Excavata, have been widely detected in deep waters while Alveolata and Stramenopiles dominate in surface waters (Scheckenbach et al., 2010; de Vargas et al., 2015; Xu et al., 2017). However, compared with surface/epipelagic waters (e.g., Bachy et al., 2011; Wu et al., 2014a,b; de Vargas et al., 2015), cold methane seeps (Takishita et al., 2007), and anoxic basins (Stoeck et al., 2003; Edgcomb V.P. et al., 2011; Orsi et al., 2012), few studies have focused on planktonic microbial eukaryotes in bathypelagic and abyssal areas. Furthermore, most of these studies were conducted over a narrow depth range where it was not possible to examine vertical distribution patterns (Countway et al., 2007; Xu et al., 2017).

Deep-sea life is heavily influenced by marine snow [particulate organic matter (POM) flux from the euphotic zone] which provides hotspots of microbial diversity and activity (Bochdansky et al., 2010). Marine snow, combined with fecal pellets from zooplankton and fish and phytodetritus from sinking phytoplankton, is important to the biological pump, transferring particulate carbon to greater depths (Turner, 2015). The composition and sinking rate of marine snow thus influence the microbial community, which is different from the surrounding water (Turner, 2002). Marine snow harbors microbes of different nutritional types, such as saprotrophy, heterotrophy, and parasitism. A recent study has shown that two saprotrophic groups, i.e., fungi and labyrinthulomycetes, dominate the biomass of bathypelagic marine snow (Bochdansky et al., 2016), indicating that eukaryotic microbes could contribute to particle solubilization and remineralization (Pernice et al., 2015b). Heterotrophic microbial eukaryotes, acting as bacterial grazers, are also important members of bathypelagic microbial communities (Pernice et al., 2015a). Parasitic dinoflagellate (MALV-II, marine alveolates group II) species were found to be wide-spread in all water types, accounting for 10–18% of total microbial eukaryotic sequences (Pernice et al., 2015b).

The microbial loop (Azam et al., 1983) exerts a major influence on patterns of carbon and nutrient fluxes in the ocean. However, our current understanding of these ecological concepts is mainly based on studies conducted in the euphotic zone with much less information from greater depths (López-García et al., 2001; Herndl et al., 2008; Nagata et al., 2010). Despite technological and methodological advancements, current investigations have provided little information on the ecological role and function of deep-sea microbial eukaryotes (Snelgrove, 1999; Danovaro et al., 2014). “Deep-sea Microbial Ecology” is a rapidly evolving field with several large investigations having been made in last two decades (Corinaldesi, 2015). While a variety of highly diverse habitats have been recently described (Jørgensen and Boetius, 2007; Bartlett, 2009; Ramirez-Llodra et al., 2010; Danovaro et al., 2014), the ecological roles of the different groups of microbial eukaryotes in the deep sea have received little attention.

The Mariana Trench, as a part of the Izu-Bonin-Mariana subduction system, is the deepest place on earth. While recent studies have revealed distinct active prokaryotic communities in the sediments of the Mariana Trench (e.g., Glud et al., 2013), there remains little information on microbial eukaryotes, especially in the water column of the Mariana Trench. Based on vertical distribution patterns of microbial communities in the Challenger Deep (e.g., Nunoura et al., 2015) and studies of microbial eukaryotes in other deep-sea areas (e.g., Pernice et al., 2015b; Xu et al., 2017), we hypothesized that (1) some picoeukaryote groups would be distributed widely across all depths; (2) distinct microbial eukaryotic community structures might exist in the abyssal or hadal zones compared with upper layers; (3) variations in community compositions would be different between size fractions. In the present study, we focused on the vertical community structure and diversity of microbial eukaryotes from the surface to the hadal layer (8727 m) at a site above the Mariana Trench. We performed Illumina MiSeq sequencing targeting the V4 region of 18S rDNA for eukaryotes of the 0.2–3 μm and >3 μm size fractions. To reveal the potential biotic relationships across water column, we explored the ecological associations among eukaryotic microbial assemblages according to their co-occurrences.

## Materials and Methods

### Samples Collection

This study was conducted at a site above the Challenger Deep of the Mariana Trench (11.38°N, 142.30°E) on an oceanography survey of south-central western Pacific Ocean in winter (GASI-02-PAC-ST-MSwin), on R/V “Dongfanghong 2” (**Supplementary Figure S1**). Seawater samples (2 L, from each Niskin bottle, prefiltered through a 200 μm pore-sized mesh) from water depths of 0, 1000, 1759, 3699, 5367 and 8727 m were collected by Niskin bottles mounted to a Seabird CTD (SBE 16plus, with a Titanium housing for maximum depth over 10,000 m. Detailed technical information^1^) on January 3rd, 2016. These sampling depths were selected based on the hydrographic conditions of the site and to reflect the microbial eukaryotic communities in the epipelagic (0–200 m), mesopelagic (200–1000 m), bathypelagic (2000–4000 m), abyssopelagic (4000–6000 m), and hadopelagic (>6000 m) zones. After collection, each sample was immediately filtered through a 3 μm pore-sized polycarbonate filter (with a gentle vacuum pressure <25 cm Hg), followed by a 0.22 μm filter (Whatman, Piscataway, NJ, United States). Samples of 0.2–3 μm from surface to the deepest layer were named 0m-0.2, 1000m-0.2, 1759m-0.2, 3699m-0.2, 5367m-0.2, and 8727m-0.2, respectively, and samples of >3 μm were named 0m-3, 1000m-3, 1759m-3, 3699m-3, 5367m-3, and 8727m-3, respectively. Each filter was carefully placed into a 5 mL tube containing 2 mL of Lysis buffer (50 mM Tris-HCl, 1.0 mM EDTA, 150 mM NaCl, and 0.1% SDS). The tubes were quickly frozen in liquid nitrogen and stored at -80°C until DNA extraction. Depth, temperature and dissolved oxygen (DO) were measured *in situ* by the sensors on the CTD equipment while nutrient concentrations (PO_4_, NO_3_, NO_2_, NH_4_, and SiO_3_) were subsequently analyzed on return with a continuous-flow auto-analyzer (AA3, Seal Analytical Inc., Southampton, United Kingdom).

### DNA Extraction and PCR Amplification

Filters were thawed and DNA extraction was performed according to Stoeck and Epstein (2003) using a phenol-chloroform-isoamyl (1:1) extraction combined with precipitation and washing procedures: proteinase K (100 μg mL^-1^) was added and incubated (55°C, 1 h); the lysates were mixed twice with phenol–chloroform–isoamyl (both 500 μL); 100% ethanol and 50 μL NaAc (1 M) were added to precipitate DNA (incubated in -20°C for 2 h); the pellet was washed with 1 mL 70% ethanol, dried and dissolved in 50 μL water (Bostrom et al., 2004). The precipitated DNA extracts were diluted in 30 μL of ddH_2_O and kept at -20°C for further analysis. For PCR amplification, bidirectional primers were designed to amplify the V4 region of 18S SSU rDNA: the forward 3NDF (5′-GGCAAGTCTGGTGCCAG-3′) and the reverse V4_euk_R2 (5′-ACGGTATCT(AG)ATC(AG)TCTTCG-3′) (Bråte et al., 2010). A subsequent limited-cycle amplification step was performed to add overhang adapters and library-specific barcodes to primers. PCR reactions were performed in a triplicated 20 μL mixture containing 4 μL of 5× FastPfu Buffer, 2 μL of 2.5 mM dNTPs, 0.8 μL of each primer (5 mM), 0.4 μL of FastPfu Polymerase and 10 ng of template DNA. PCR amplification was completed at 95°C for 2 min, followed by 35 cycles of 95°C for 30 s, 55°C for 30 s, 72°C for 45 s, and a final extension of 10 min at 72°C.

### Illumina MiSeq Sequencing and Data Processing

PCR products were checked by 2% agarose gel electrophoresis and purified using the AxyPrep DNA Gel Extraction Kit (Axygen Biosciences, Union City, CA, United States) and quantified using QuantiFluor^TM^-ST (Promega, United States). The concentrations of these purified DNA extracts were measured with a Qubit 2.0 fluorometer (Thermo Fisher Scientific Inc., United States). The purified products were then pooled in equimolar concentrations for paired-end sequencing on an Illumina MiSeq PE300 platform^2^. Raw reads in fastq files with low quality (Q < 20 or length <200 bp) were discarded using QIIME (Version 1.17) (Caporaso et al., 2008). Tags were obtained by merging the paired reads according to their overlaps, using COPE (Connecting Overlapped Pair-End, V1.2.3.3) (Liu et al., 2012), after cutting off the sequences of barcodes and primers. High quality pair-wise sequences were obtained following these standards: (i) bases with ASCII value below 33 were screened out; (ii) a minimum overlap of 19 bp between reads; (iii) no more than one mismatch was accepted while cutting off the sequences of primers. Operational taxonomic unit (OTU) clustering was performed at a minimum sequence similarity of 97% using UPARSE (Edgar, 2013). Chimeric sequences were screened out through UCHIME (Edgar et al., 2011). Representative sequences of each OTU were assigned using the Silva (SSU115) 18S rRNA database (Quast et al., 2013) based on a confidence threshold of 70% for taxonomic affiliations. Non-affiliated OTUs, OTUs affiliated with Archaea and terrestrial plants and singletons were removed from the data set. Sequence data generated in this study have been deposited in the NCBI Sequence Read Archive (SRA) under BioProject PRJNA399026.

### Statistical and Phylogenetic Analyses

R software (version 3.4.1^3^) was employed to compute diversity and richness indices with the “Vegan” package (Oksanen et al., 2016). Alpha diversity (OTU richness), Chao1 and Shannon indices were calculated based on the standardization of the sample with lowest sequences (*N* = 12,889 sequences). Abundant OTUs and groups, with sequence proportion over 1% at a given site, were focused on in this study (Logares et al., 2014). The taxonomic affiliations of eukaryotes, of both super group and lower group level, were determined following Worden et al. (2015). Super groups mainly include Archaeplastida, Amoebozoa, Opisthokonta, Excavata, Rhizaria, Alveolata, Stramenopiles, and lower groups include some sub-taxonomies at the phylum or class level. A maximum likelihood (ML) phylogenetic tree of abundant OTUs and reference species was built (bootstrap replications = 1000) after alignment of their sequences using MEGA v7.0 (Kumar et al., 2008). A heatmap of locally abundant OTUs was generated by the “pheatmap” package in R with corresponding taxonomy to each OTU (Kolde, 2015). Non-metric multidimensional scaling (nMDS) analysis was performed with PRIMER v6.1 (Clarke and Gorley, 2006), using square root-transformed sequence data. Differences among groups discriminated by nMDS were tested using permutational multivariate analysis of variance (PERMANOVA) (Anderson et al., 2008). Co-occurrence of pairwise OTUs within and among groups was analyzed and depicted by employing “cooccur” and “circlize” packages in the R software using presence/absence data (Lima-Mendez et al., 2015). Based on the results of the detrended correspondence analysis (DCA), redundancy analysis (RDA), and canonical correspondence analysis (CCA) were used for constrained analyses between environmental variables and microbial eukaryotic communities (by “Vegan” package in R 3.4.1). Environmental factors most related with variation of community changes were selected in this correlation analysis by using the “bioenv” function in “Vegan” package (with significance tested by the “envfit” function, permutation = 999).

## Results

### Environmental Factors and Relationships With Microbial Eukaryotic Communities

The vertical temperature and salinity profiles were similar to previous report (Taira et al., 2004, 2005; Nunoura et al., 2015) (**Table 1**). The highest DO concentration was observed at the surface (205.22 μM) and the lowest was found at a depth of 1000 m (86.48 μM). The pH was highest at the surface (8.3) and remained at around 7.9 at all other depths. The nitrate (NO_3_) concentration was low at the surface (0.4 μM) but increased strongly with depth, sharing a similar pattern with phosphate, silicate and dissolved inorganic nitrogen (DIN) distributions. The ratios of nitrogen to phosphorus (N/P) used here were estimated by dividing DIN by the PO_4_ concentration; the highest N/P ratio occurred at the surface (19.23).

**Table 1:** Environmental factor values of different depths of sampling site.

Depth (m)	Tem (°C)	Sal	DO (μmol/L)	pH	PO_4_ (μmol/L)	NO_3_ (μmol/L)	NO_2_ (μmol/L)	NH_4_ (μmol/L)	SiO_3_ (μmol/L)	DIN	N/P
0	28.10	34.30	205.22	8.3	0.12	0.40	0.06	1.81	1.36	2.27	19.23
1000	4.34	34.55	86.48	7.85	2.84	41.47	0.05	0.72	109.22	42.24	14.86
1759	2.3	34.60	121.95	7.89	2.65	38.10	0.04	1.19	139.81	39.34	14.84
3699	1.49	34.66	159.19	7.93	2.54	27.73	0.03	1.33	161.65	29.09	11.46
5367	1.50	34.68	180.94	7.92	2.42	43.10	0.02	0.48	150.00	43.60	18.05
7200	1.75	34.69	NA	NA	2.43	31.28	0.03	0.43	150.00	31.74	13.06
8727	2.00	34.68	173.24	7.82	2.43	35.30	0.06	0.61	142.72	35.97	14.80

*Tem, temperature; Sal, salinity; DO, dissolved oxygen; DIN, dissolved inorganic nitrogen; N/P, ratio of nitrogen to phosphorus; NA, means data is not available.*

Because of strong vertical gradients in most environmental factors (≥1000 m) (e.g., silicate concentration, 1.36 μmol/L at the surface but over 100 μmol/L in deep waters), the relationships between environmental factors and the samples were analyzed with and without surface layer values (**Supplementary Figure S2**). With surface samples included, the 0.2–3 μm communities were significantly influenced by DO (*p* = 0.034), silicate concentration (*p* = 0.008) and salinity (*p* = 0.019), while the >3 μm communities were significantly influenced by DO (*p* = 0.026). After excluding the surface samples, community variations in the 0.2–3 μm and >3 μm fractions were significantly related to both DO (*p* = 0.033 and 0.033, respectively) and phosphate concentration (*p* = 0.033 and 0.008, respectively).

### Alpha Diversity

After the removal of low quality reads, a total of 436,160 sequences and 910 OTUs (0.2–3 μm, 636 OTUs; >3 μm, 780 OTUs) were obtained from protist (unicellular eukaryotes) taxa (**Table 2**). The rarefaction curves of the samples’ OTU richness were sampled to near saturation, indicating that the sequencing effort had exhaustively sampled the microbial eukaryotic diversity at each depth (**Supplementary Figure S3**). OTU richness was highest in the >3 μm fraction of the surface (431 OTUs) while the lowest OTU richness appeared at the greatest depth with only 84 OTUs. Notably, within each depth, OTU richness in >3 μm sample was higher than that in 0.2–3 μm sample. Diversity index Chao and Shannon showed the same trend.

**Table 2:** Contextual data of 12 samples in this study.

Sample	Size (μm)	Depth (m)	Sequences	OTUs	Chao	Shannon
0m-0.2	0.2–3	0	32,047	380	427	2.86
0m-3	>3	0	25,875	431	475	4.23
1000m-0.2	0.2–3	1000	31,847	157	179	2.93
1000m-3	>3	1000	29,095	256	318	2.2
1759m-0.2	0.2–3	1759	32,390	227	267	2.98
1759m-3	>3	1759	28,093	284	343	2.79
3699m-0.2	0.2–3	3699	38,095	105	115	1.2
3699m-3	>3	3699	29,640	228	250	3.53
5367m-0.2	0.2–3	5367	34,833	139	155	3.38
5367m-3	>3	5367	27,890	314	343	4.22
8727m-0.2	0.2–3	8727	17,131	84	112	1.99
8727m-3	>3	8727	12,889	145	183	1.58
Total sequences			436,160			
Total unique OTUs				910		

*Only protistan sequences and OTUs were included.*

Metazoan taxa comprised 18.5% of the total sequences (44 OTUs) in the 0.2–3 μm fraction and 24.2% of the total sequences (38 OTUs) in the >3 μm fraction. Most of the Metazoan sequences were affiliated to Cnidaria, Arthropoda, and Vertebrata. Generally, Cnidaria sequences dominated the Metazoan sequences from surface layer to 1759 m, while Vertebrata sequences were the most abundant at depth over 3699 m. As most sequences may have come from either dead bodies (which can be important food resources for microbial communities in the marine snow) or extracellular DNA, and regarding the high number of 18S rDNA copies in the Metazoan cells, they were not further analyzed based on the proportion of sequences (only included in the co-occurrence analysis where presence and absence data were used).

### Community Structures and Abundant OTUs

**FIGURE 1:**
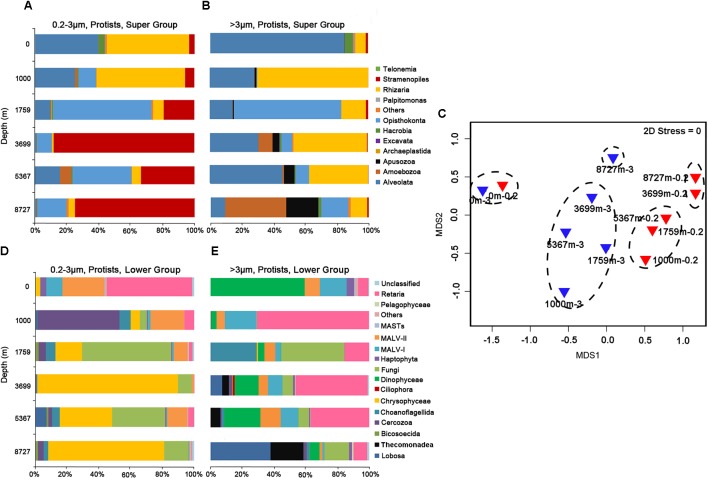
Sequence proportions of taxonomic groups in the **(A)** 0.2–3 μm fraction at the super group level; **(B)** >3 μm fraction at the super group level; **(D)** 0.2–3 μm fraction at lower group level; **(E)** >3 μm fraction at lower group level. Only protistan sequences were included. **(C)** Shows the grouping of samples in the non-metric multidimensional scaling (nMDS) by community similarities, using Bray–Curtis distance.

The sequence proportion of eukaryotic microbial groups, at super group and lower level (mainly phylum or class) were calculated for each sample to show the community structure at each depth (**Figure 1**). Community composition of microbial eukaryotes differed with depth. For the 0.2–3 μm fraction, at the super group taxonomic level (**Figure 1A**), sequences of Alveolata and Rhizaria dominated in the surface and 1000 m samples. Opisthokonta sequences were abundant at 1795 m (61.8%). Sequences belonging to Stramenopiles were abundant at 3699 m (65.6%) and greater depths. At lower group levels (**Figure 1D**), sequences of Retaria and MALV-II dominated at the surface (with 53.5 and 26.3%, respectively). The community at 1000 m was mainly composed of Cercozoa (51.2%) and MALV-II (21.2%). Fungi dominated at 1759 and 5367 m depths with sequence proportion of 56.8 and 33.2%, respectively. Both the 3699 and 8727 m samples were overwhelmingly dominated by sequences of Chrysophyceae (80.5%, on average).

For protistan sequences in the >3 μm fraction, at the super group level (**Figure 1B**), Alveolata sequences overwhelmingly dominated the surface sample (85%). Rhizaria sequences were most abundant at 1000 m (69.9%). Opisthokonta sequences accounted for 65% of the total sequences at 1759 m. Alveolata and Rhizaria sequences dominated at 3699 and 5367 m while Amoebozoa and Apusozoa sequences were most abundant at 8727 m (38 and 20.4%, respectively). At the lower taxonomic levels (**Figure 1E**), Dinophyceae sequences dominated the surface community (49.3%). Retaria sequences were found in all samples with an average proportion of 30.9%. Fungi sequences were most abundant at 1000 m (40%). Sequences of Lobosa within Amoebozoa dominated the community at 8727 m, accounting for 39.5% of total sequences.

Hierarchical clustering based on SIMPROF divided the 12 samples into five groups (Group 1: 0m-0.2 and 0m-3; Group 2: 1000m-0.2, 1759m-0.2 and 5367m-0.2; Group 3: 3699m-0.2 and 8727m-0.2; Group 4: 1000m-3, 1759m-3, 3699m-3 and 5367m-3; Group 5: 8727m-3) at a similarity level of 35%. This grouping result is presented on the nMDS map (**Figure 1C**), where difference between each two groups (Groups 1–5) is statistically significant (*p* < 0.05, PERMANOVA test). The average dissimilarity (Bray–Curtis) between the samples in the larger size fraction (54.8%) is greater than that in the 0.2–3 μm fraction (49.4%).

**FIGURE 2:**
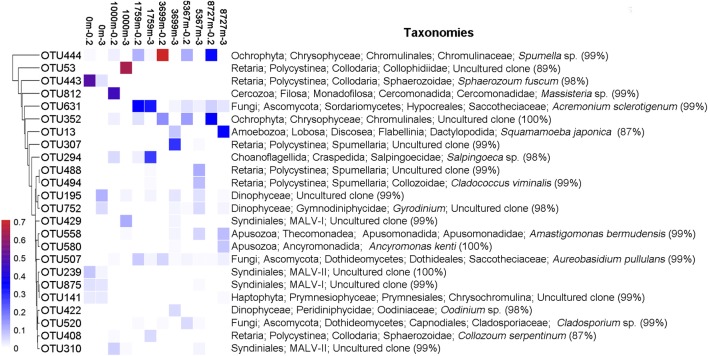
Heatmap of abundant OTUs of the 12 samples. Abundant OTU refers to OTU with sequence proportion over 1% in a given sample. The corresponding taxonomy of each OTU is on the right, with the sequence similarity to the nearest match.

For the locally abundant OTUs (sequence proportion over 1% in a given sample) in the two fractions (**Figure 2**), it was found that sequences of OTU444 and OTU352 (both belonging to Chrysophyceae) dominated in the 0.2–3 μm fraction with depths ≥1759 m (averagely 32.1 and 17.5%, respectively), while sequences of OTU195 and OTU752 (both belonging to Dinophyceae) were abundant in the >3 μm fraction from the surface to the deepest layer (averagely 4.1 and 2.5%, respectively). Sequences of OTU631 and OTU507, within Fungi, had wide distributions in both fractions at depths ≥1000 m (averagely 8.8 and 2.4%, respectively). Sequences of OTU812 (*Massisteria* sp., Cercozoa) was highly enriched in sample 1000m-0.2 (46.5%), but was uncommon in other samples (0.1%, on average).

**FIGURE 3:**
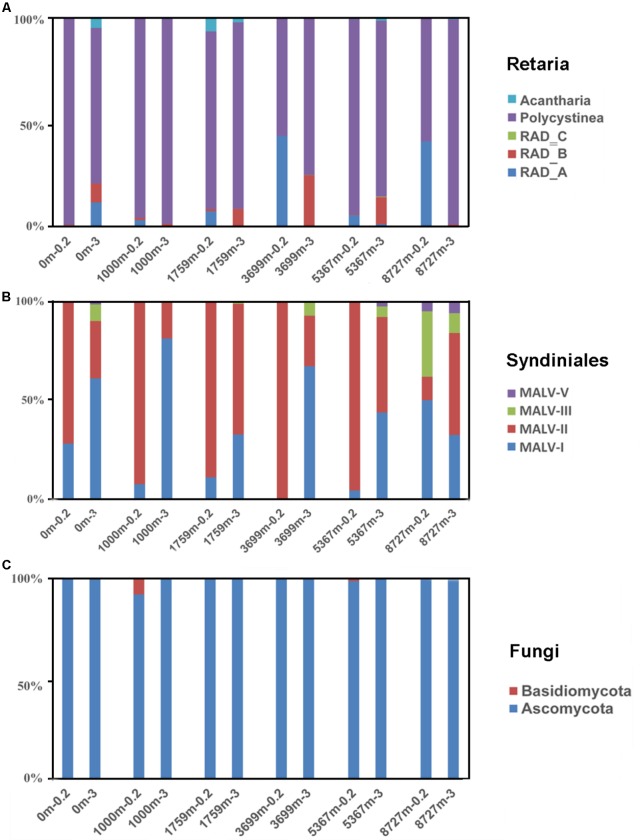
Sequence proportions of sub-taxonomies within **(A)** Retaria, **(B)** Syndiniales, and **(C)** Fungi. Sub-taxonomies with no sequences in the samples were not shown.

Retaria, Syndiniales (mainly MALVs) and Fungi were widely distributed in all samples with abundant sequences and high OTU richness (on average 14.1, 36.6 and 8.9% of the OTU richness proportion in a single sample, respectively). Their compositions of sub-taxonomies are shown in **Figure 3** (Sub-taxonomies with no sequences in this study are not shown). Generally, Polycystinea dominated the Retaria sequences in both fractions (84.4%, on average). Within Polycystinea, the order Collodaria (represented by OTU443, OTU408, and OTU53) was abundant in upper layers (0–1759 m), while the order Spumellaria, close relatives of heterotrophic grazers, (represented by OTU494, OTU307, and OTU488) was enriched in deeper waters (≥3699 m) (**Supplementary Figure S4**). Within Syndiniales, MALV-I and MALV-II sequences were most abundant (on average 35 and 58% of the total Syndiniales sequences, respectively) in all samples, Ascomycota overwhelmingly dominated the Fungi sequences (99.3%, on average), compared with Basidiomycota.

### Associations Between Groups

**FIGURE 4:**
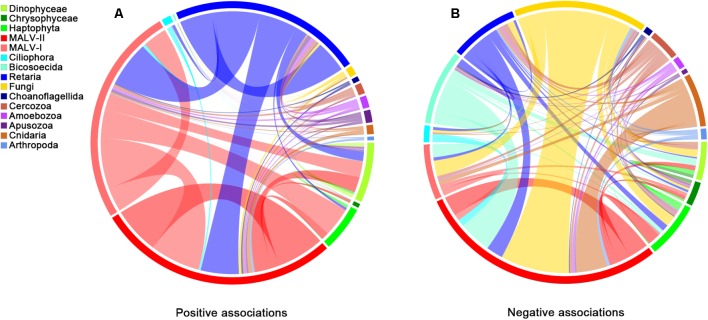
Co-occurrence of different groups based on network analysis. Ribbons connecting two segments indicate co-presence **(A)** and exclusion **(B)** links, on the left and right. Size of the ribbon is proportional to the number of links (co-presence and exclusion) between OTUs assigned to the respective groups. Links are dominated by Retaria, MALV-I and MALV-II in the positive associations (Left) and by Retaria and Fungi in the negative associations (Right).

According to the co-occurrence analysis, a total of 3145 pairwise OTUs were found with significantly positive associations (**Figure 4A**). MALV-I, MALV-II and Retaria showed the greatest contributions to the relationships within and between groups (60.8%). Positive co-occurrence was also found for the above three groups and others, for instance, between MALV-I and Haptophyta (7.4% of total). For the negative associations (**Figure 4B**), a substantial number of pairwise OTUs (966, in total) were found within and between groups as well. Pairwise OTUs between Fungi and MALV-II represented 17.5% of the total negative associations.

## Discussion

Despite recent methodological and technological advances, it is estimated that only 5% of the deep oceans has so far been explored and that less than 0.001% has been sampled and described in terms of biodiversity (Danovaro et al., 2014; Corinaldesi, 2015); even less is known about microbial eukaryotes. Furthermore, many microorganisms are fragile and may have regularly been lost during filtering, making their diversity under-presented and poorly understood (Biard et al., 2016). The filtration procedures used here were performed on board the ship at room temperature. The increase in seawater temperature after retrieval may have influenced the documented microbial eukaryotic community. However, this effect will likely be minor for microorganisms examined at the DNA level.

In this study, the species richness of microbial eukaryotes was highest at the surface and lowest at the bottom, 8727 m (for both size fractions), suggesting that great differences in the living environment of microbial eukaryotes may exist in the hadal zone, compared with upper layers (Jamieson et al., 2010). In addition, a comparison of OTU richness of the two size fractions at each depth showed that microbial eukaryotes were more diverse in the >3 μm size fraction than in the 0.2–3 μm fraction, which was consistent with the results of Parris et al. (2014).

### Abundant Groups Represented by Retaria, MAVLs, and Fungi

Marine microbial eukaryotic communities are usually composed of a few of locally abundant species and many rare species. The abundant species or groups dominate the community not only by having large numbers of individuals but also by performing the major ecosystem functions (Logares et al., 2014). It is worth commenting, however, that the number of 18S rDNA sequences does not represent cell abundance. Recent studies have shown that the number of rDNA copies in protists varies greatly, from a few in picoeukaryotes to more than thousands in larger sized protists, such as dinoflagellates and ciliates (e.g., Zhu et al., 2005; Gong et al., 2013). Moreover, a recent study using single-cell approach to quantify ribotype copy numbers in ciliates found that the sequence number of 18S rDNA is more closely related to a population’s biomass than cell abundance (Fu and Gong, 2017). Although this difference in gene copy numbers can be reduced by analyzing the size fractions separately, data derived from rDNA sequencing (e.g., HTS) needs to be interpreted with caution.

In this study, a number of abundant groups and species were identified that characterized differences between communities at different depths and between different size fractions. While the super groups SAR (i.e., Stramenopiles, Alveolata, and Rhizaria) and Archaeplastida have been widely reported as the dominant groups in various surface waters (Massana, 2011), studies of the vertical distributions of abundant microbial eukaryotes from surface to the deep water remain scarce. In a recent study by Pernice et al. (2015b), Collodaria, Chrysophyta, Fungi, and MALV-II were found to be dominant in the bathypelagic waters at 27 stations in the Atlantic, Pacific, and Indian Oceans. In addition to these four groups, large numbers of sequences affiliated to Amoebozoa and Apusozoa were also found in the deep-sea samples studied herein. The relative proportions of these abundant groups, however, varied among samples of different depths, showing a high level of variability.

Sequences of Rhizaria, which were all affiliated to Radiolaria (amoeboid protists belonging to Retaria) were abundant at all depths in this study. Radiolarians, comprised of five orders (i.e., Acantharia, Taxopodia, Collodaria, Nassellaria, and Spumellaria), are thought to actively contribute to the deep-sea heterotrophic communities, especially in the mesopelagic and bathypelagic zones. Furthermore, Acantharia species play an important role in the biological pump of carbon by contributing to the deep-sea particulate organic carbon (POC) flux via cyst formation (Bernstein et al., 1987; Martin et al., 2010). Radiolarian sequences from the surface layer were mostly represented by OTU443 (98.1% of the total Retaria sequences) with a 98% identity to *Sphaerozoum fuscum* (Collodaria), which was recently characterized as a colonial species living in the photic zone (Biard et al., 2017). In addition to the PCR bias, the enrichment of radiolarians at the surface in the 0.2–3 μm fraction might have been derived from extracellular material of larger radiolarian cells (Not et al., 2009) or small reproductive cells (gametes) (Not et al., 2007).

In the >3 μm fraction, the class Polycystinea (including Collodaria and Spumellaria) overwhelmingly dominated the Retaria sequences (91.16%), consistent with previous studies (Pernice et al., 2015b; Xu et al., 2017). Notably, the most abundant Retaria OTU at each depth was affiliated to a different order. As the most abundant Retaria OTUs at 1000 and 1759 m, respectively, OTU53 and OTU408 belonged to the order Collodaria (with the nearest sequence match to the uncultured clone from pelagic water of South China Sea, accession number KX532676.1), while the most abundant OTUs at depths ≥3699 m belonged to the order Spumellaria. Moreover, Collodaria comprised 76% (on average) of the total Retaria sequences at depth ≤1759 m, while Spumellaria comprised 85.7% (on average) at depths ≥3699 m, suggesting a difference in depth preference for the two orders. A previous study has found that some radiolarians living at the surface can produce cysts that sink rapidly and release flagellated cells at depth (Decelle et al., 2013) and it is thought that this may have led to the uneven distributions of radiolarians in the deep sea. Although the presence of DNA sequences of radiolarians does not infer their activity, the possibility that some radiolarians live in the deep sea should not be discounted.

As one of the most phylogenetically diverse groups within Alveolata, MALV (marine alveolates) has frequently been reported from its uncultured parasitic sub-groups MALV-I and MALV-II (Massana, 2011). MALV-II, also known as Syndiniales Group II, was widespread, including in the deep ocean (Guillou et al., 2008; Pernice et al., 2015b). It comprised 18.2 and 11.7% of the picoeukaryotic (0.2–3 μm) richness and sequence abundance, respectively, in the present study. An average of 6.2% of the total sequences in >3 μm fraction also belonged to MALV-II, indicating that potential hosts affected by MALV-II parasites may exist in this size fraction, especially dinoflagellates. Among MALV-II, *Amoebophrya* spp. are host specific species infecting different free-living dinoflagellates (Coats and Park, 2002). They were also found in the present study with 76 OTUs, none of which were among the abundant OTUs. However, MALV-I appeared to make up a greater proportion of sequences in the >3 μm fraction than MALV-II at surface and 1000 m, suggesting that it has a wider host spectrum, especially in the lager sized fraction, even in the deep sea (Massana, 2011).

The widespread distribution of MALVs (abundant throughout the water column) in the samples investigated herein suggests that microbial eukaryotic heterotrophy in the deep sea is largely represented in the form of parasitism (Pernice et al., 2015b). Many parasitic groups, e.g., *Amoebophrya* spp., are host-specific; however, whether the specific parasitism exists consistently in the deep sea has received little attentions so far. The network of interactions here demonstrated consistent biotic relationships within and between microbial eukaryotic groups throughout the water column. Positive associations, mainly represented by MALV-I, MALV-II, and Retaria, outnumbered negative associations by a ratio of 3:1, which is similar to that found in the photic zone (Lima-Mendez et al., 2015), suggesting wide distributions of parasitism throughout the water column. Syndiniales are an order of dinoflagellates, found exclusively as endosymbionts (Chambouvet et al., 2008). Some groups with affiliation to Syndiniales, e.g., MALV-II, can parasitize plankton such as Phaeodarea (Cercozoa), Acantharea (Radiolaria), and Polycystinea (Radiolaria) (Guillou et al., 2008) and have a widespread distribution in the deep sea (Not et al., 2007). Moreover, individuals of MALV-I and MALV-II have been directly isolated from the cells of Radiolaria (Dolven et al., 2007), supporting the likely parasitism between Syndiniales and Retaria in deep waters. Species within MALV-I also have widespread distributions and host spectrums (Guillou et al., 2008) and in this study, seem to infect Dinophyceae and Haptophyta, based on positive associations among these groups. MALV-I and MALV-II might, however, have the same hosts and infect them simultaneously, as few negative associations were found between them.

Fungi are osmotrophs, feeding by externally processing nutrients into metabolites (Richards et al., 2015). Marine fungi have been reported to be one of the main component of marine snow (Bochdansky et al., 2016) and Basidiomycota, within Fungi, is the dominant microbial eukaryotic group in the deep sea (3000–4000 m) worldwide (Pernice et al., 2015b). They were found to be the most abundant taxa in the hadal waters of the Puerto Rico Trench (Eloe et al., 2011). In this study, Fungi were most abundant at 1759 m. Ascomycota was much more abundant than Basidiomycota in terms of both sequence number and richness, suggesting that the composition of this group may be quite different in some areas, even though the physical environment was relatively uniform (Junior et al., 2015). Fungi are largely responsible for the decomposition of organic matter in the deep sea and previous studies have shown that Fungi are capable of the degradation and utilization of refractory organic material that other microorganisms cannot use (Clipson et al., 2006). It is possible that they stabilize the composition of marine snow, in a similar manner to their contribution in soils (Chenu and Stotzky, 2002). The mutual exclusions between MALV-II and Fungi here might indicate that saprophytism (represented by Fungi) is more favored in the deep sea than parasitism (represented by MALV-II), as more refractory organic material (or dead organism bodies) and fewer potential hosts remain in the marine snow at greater depths.

The grouping of the two surface samples together was due partly to the similar contributions of different abundant groups (especially some phototrophs) to the community structures, which is similar to the community patterns found in the water columns of northern Chile (Parris et al., 2014) and the South China Sea (Xu et al., 2017). While Retaria was the most abundant group at surface in the 0.2–3 μm fraction, Dinophyceae dominated the surface sample in the >3 μm fraction and was also present at all other depths. Many Dinophyceae species (e.g., species within *Gymnodinium*) produce cysts, which can sink into deep waters, as part of their life cycle (Bravo and Figueroa, 2014). Since the deep water enriched Dinophyceae OTUs (e.g., OTU195 and OTU752, both belonged to *Gymnodinium*) also had abundant distributions at the surface, the presence of Dinophyceae sequences at greater depths (≥1000 m) could be attributed to sinking cysts (Gisselson et al., 2002).

### Hadal Zone Communities

Microbial communities (including both prokaryotes and eukaryotes) can be grouped (e.g., UPGMA clustering) by their depth in the water column, possibly reflecting their adaptation to pressure (Brown et al., 2009). Communities of prokaryotes (both bacteria and archaea) in the hadal waters were found to be distinct from those in abyssal waters, suggesting that the unique hadal biosphere in the Challenger Deep may be strongly influenced by the input of organic matter followed by heterotrophic degradation (Nunoura et al., 2015). However, little is known about the differences between the microbial eukaryotic community structures of the hadal waters and upper layers. Thus, a deeper understanding of the microbial eukaryotic community composition of the hadal zone is necessary to identify the main activities and contributions of microbial eukaryotes to the carbon pump, nutrient cycling and energy transfer at extreme depth (Corinaldesi, 2015). The present study compared the community structures of microbial eukaryotes along a depth gradient to the deep sea, with hadal samples included for the first time.

While previous studies have reported a wide variety of metazoan species (mainly benthic fauna) (Jamieson et al., 2010) in trenches, planktonic protists have only rarely been studied. In this study, the grouping of the samples 3699m-0.2 and 8727m-0.2 in the nMDS analysis was mainly due to the overwhelming dominance of Chrysophyceae (78.3%, on average), together with the low overall species richness (eight OTUs, in total). This was similar to the prokaryotic community pattern found in the hadopelagic water of the Mediterranean Sea, where the diversity was extremely low and a “deep-ecotype” of *Alteromonas macleodii* overwhelmingly dominated the community (Smedile et al., 2013). As a major photosynthetic group (Vaulot et al., 2008), Chrysophyceae contains many mixotrophic species (Holen and Boraas, 1996) and many cultured species, such as *Spumella* sp. and *Ochromonas* sp., are known to be bacterial grazers (Rønn et al., 2002; Pfandl et al., 2004). In the present study, sequences belonging to Chrysophyceae comprised only 2.7% of the surface sample but up to 43% of the deeper samples (≥1000 m) in the 0.2–3 μm fraction. As the most abundant OTU within Chrysophyceae, OTU444 (68.01% of the total Chrysophyceae sequences), showed a 99% identity with the sequence of *Spumella* sp. (accession number: KF651119.1), which could potentially also be a heterotrophic species and thus play an active role in this deep-sea ecosystem (Pernice et al., 2015b). Similarly, OTU352 as the second most abundant Chrysophyceae species (31.5% of the total Chrysophyceae sequences), was also affiliated to the genus *Spumella*. Thus, heterotrophic Chrysophyceae species may be some of the most important grazers in the deep sea.

Amoebozoa groups, which employ phagocytosis, are important bacterial grazers and have usually been reported from sediments (e.g., Quaiser et al., 2011; Lesniewski et al., 2012). However, earlier studies have shown that Amoebozoa also has a substantial presence in both the deep Pacific Ocean (from 500 to 3000 m) (Sauvadet et al., 2010) and the Southern Ocean (170 m) (Bachy et al., 2011). The most abundant Amoebozoa species (OTU13) found at 8727 m had a closest sequence match to *Squamamoeba japonica* (87%), which was isolated from the sediment (2700 m) of the Sea of Japan (Kudryavtsev and Pawlowski, 2013). It might represent a novel unknown Amoebozoa species living in hadal waters. The heterotrophic grazers, Apusozoa species, which have frequently been detected in fresh water, cold seeps, and hydrothermal sediments, show adaptations to a wide range of salinity, temperature, and oxygen conditions (Torruella et al., 2017). However, the distributions of Apusozoa, especially *Amastigomonas bermudensis* and *Ancyromonas kenti* (the two most abundant Apusozoa species in this study, represented by OTU558 and OTU580, respectively), in the hadal water have not been studied so far.

Cell size has an important influence on the ability of organisms to adapt to environmental changes (Toigo et al., 2006). Many tiny organisms (e.g., bacteria and protists with cell size <3 μm) inhabit marine snow aggregates (with size >3 μm) and the abundances of these attached microorganisms can be much higher than in the adjacent water column (Kiørboe, 2003). A previous study conducted in a pelagic trench (6000 m) showed that microbial (both bacteria and archaea) diversity was higher in the >3 μm fraction (particle-associated) than in the 0.2–3 μm fraction (free-living), coupled with significant compositional differences between the two size fractions (Eloe et al., 2011). In this study, the diversity of microbial eukaryotes was mostly higher in the >3 μm fraction than in the 0.2–3 μm fraction and the community structure of the hadal sample, 8727m-3,was significantly different from those of the upper layers (compared to the much smaller difference in the 0.2–3 μm fraction between the community of hadal sample and the community at 3699 m), supporting the view that differences in protistan communities were related more to the size fractions, than to sample depth (Sauvadet et al., 2010).

## Conclusion

In this study, two size classes of microbial eukaryotes from the surface to the hadal zone of the Mariana Trench were examined. Some abundant groups, such as radiolarians, Dinophyceae and Fungi, had widespread distributions across all depths and showed heterogeneous relationships to depth and size fraction. Potential parasitic relationships between MALVs and Retaria were reflected by their positive associations, while mutual exclusions were suggested between MALV-II and Fungi, which could be caused by competition for food resources. This is the first record of Amoebozoa and Apusozoa as abundant groups in the hadal zone of the Mariana Trench. Together with Chrysophyceae, they could be the dominant heterotrophic grazers in the deep sea. With higher OTU richness and more variations in the community structures in the larger size fraction, especially in the hadal zone, our results suggested that size-fractionated differences should be considered when investigating the adaptation of microorganisms to the deep sea.

## Author Contributions

MW and ZX designed this study. QL and ZX performed the experiments. Data were analyzed by ZX in collaboration with YJ, YH, and YL. ZX wrote the manuscript. AM, YJ, HL, WW, and HS contributed to writing by providing suggestions and helping in revision. All authors reviewed and approved the final version of the manuscript.

## Conflict of Interest Statement

The authors declare that the research was conducted in the absence of any commercial or financial relationships that could be construed as a potential conflict of interest.

## Footnotes

1 http://archive.ssc-ras.ru/eg/Equipment/SBE_19plusV2/website/products/spec_sheets/16plusdata.htm

2 https://www.illumina.com/products/by-system/miseq-products.html

3 http://cran.r-project.org

## Supplementary Material

FIGURE S1 Location of sampling site. The station locates at a site (11.38°N, 142.30°E) above the Challenger Deep of the Mariana Trench, indicated with a red triangle in the figure.

FIGURE S2 Analysis of the relationships between microbial eukaryotic communities and environmental factors. Samples with and without surface layers were used to conduct the analysis due to the great difference of environmental factors between surface water and deeper layers (≥1000 m). Only shown were the factors most related with the community compositional changes (redundant factors were not shown).

FIGURE S3 Rarefaction curves for samples in this study. Left half of a sample name refers to depth and right half refers to size fraction (0.2 refers to 0.2–3 μm while 3 refers to >3 μm). The curve near to saturation indicates that the sequencing effort has exhaustively sampled the diversity. Metazoan sequences are included here.

FIGURE S4 Maximum likelihood phylogenetic tree of abundant OTUs in this study. Names in bold correspond to abundant OTUs found in this study while other names (in regular) refer to the nearest cultivated species or uncultured clones with accession numbers on the left (related deep-sea species were underlined). Bootstrap values above 80% are indicated by red solid circles (larger sizes represent higher values) near the nodes. Their taxonomic affiliations were shown on the right at the super group and lower group level (more specific for Rhizaria).
