# Supplementary figures and images for: Vertical Distribution of Microbial Eukaryotes From Surface to the Hadal Zone of the Mariana Trench
Source: Front Microbiol. 2018 Aug 28;9:2023. doi: 10.3389/fmicb.2018.02023 (PMC6120995; doi:10.3389/fmicb.2018.02023)

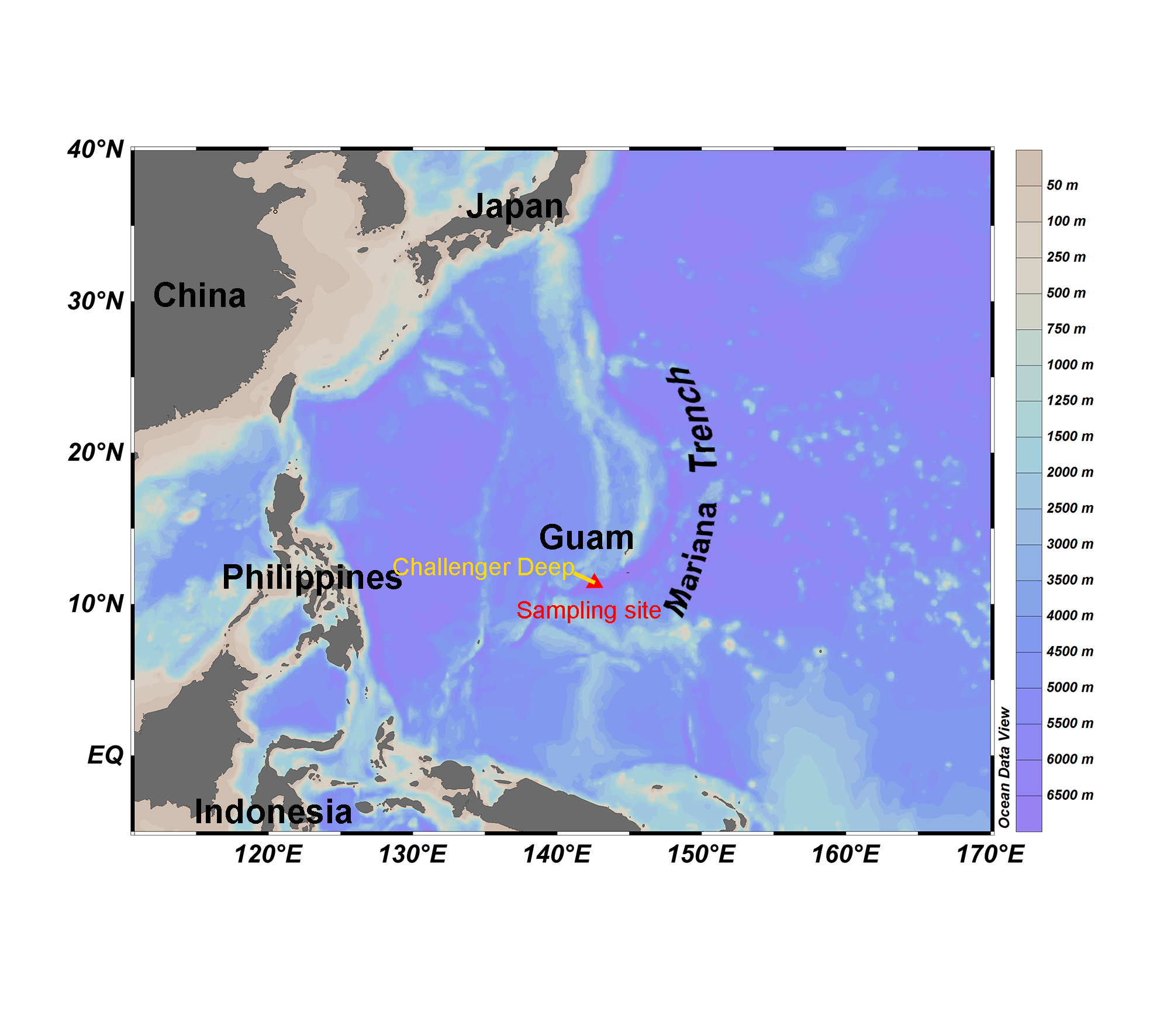

Supplement: FIGURE S1 — Location of sampling site. The station locates at a site (11.38°N, 142.30°E) above the Challenger Deep of the Mariana Trench, indicated with a red triangle in the figure. [file Image_1.TIF]

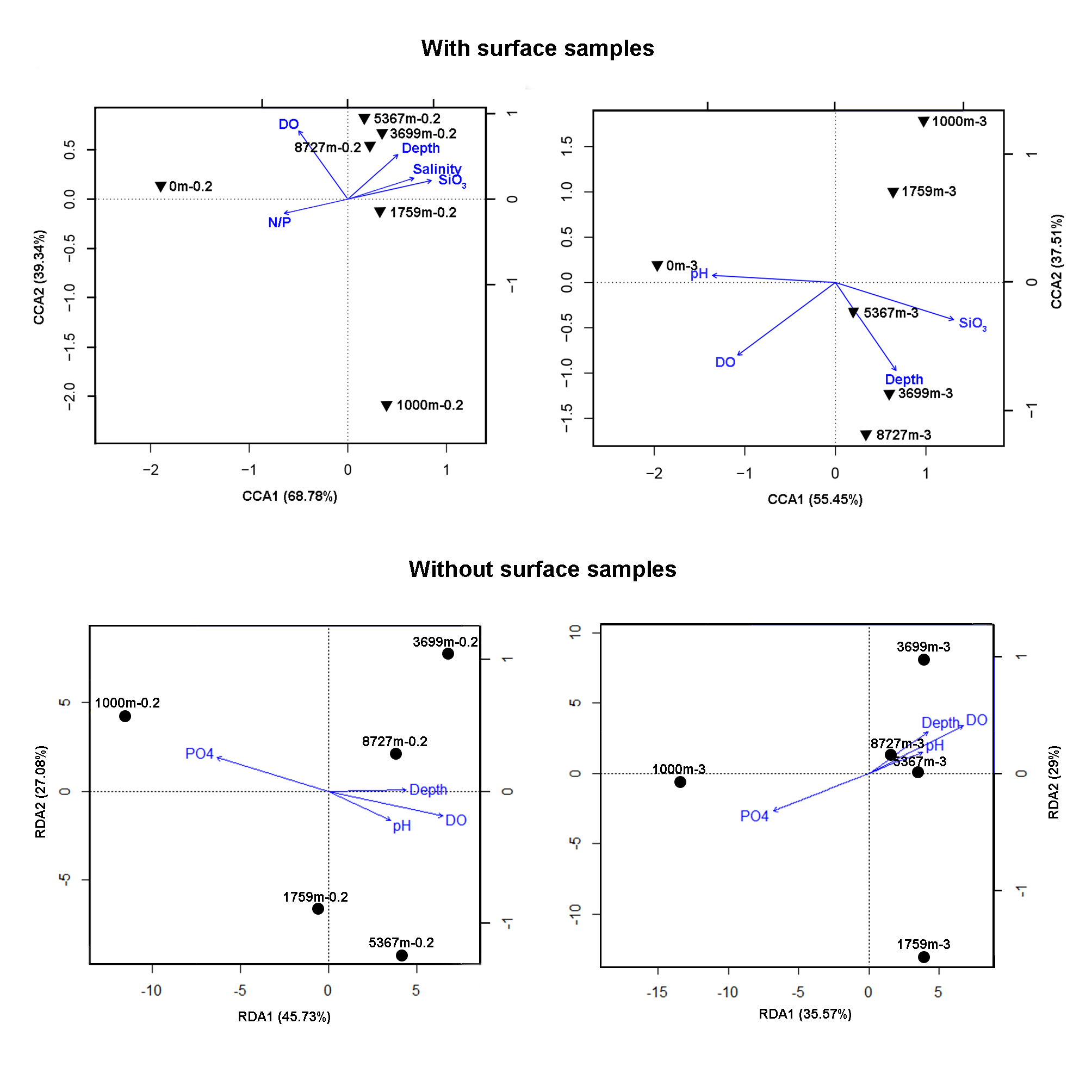

Supplement: FIGURE S2 — Analysis of the relationships between microbial eukaryotic communities and environmental factors. Samples with and without surface layers were used to conduct the analysis due to the great difference of environmental factors between surface water and deeper layers (≥1000 m). Only shown were the factors most related with the community compositional changes (redundant factors were not shown). [file Image_2.TIF]

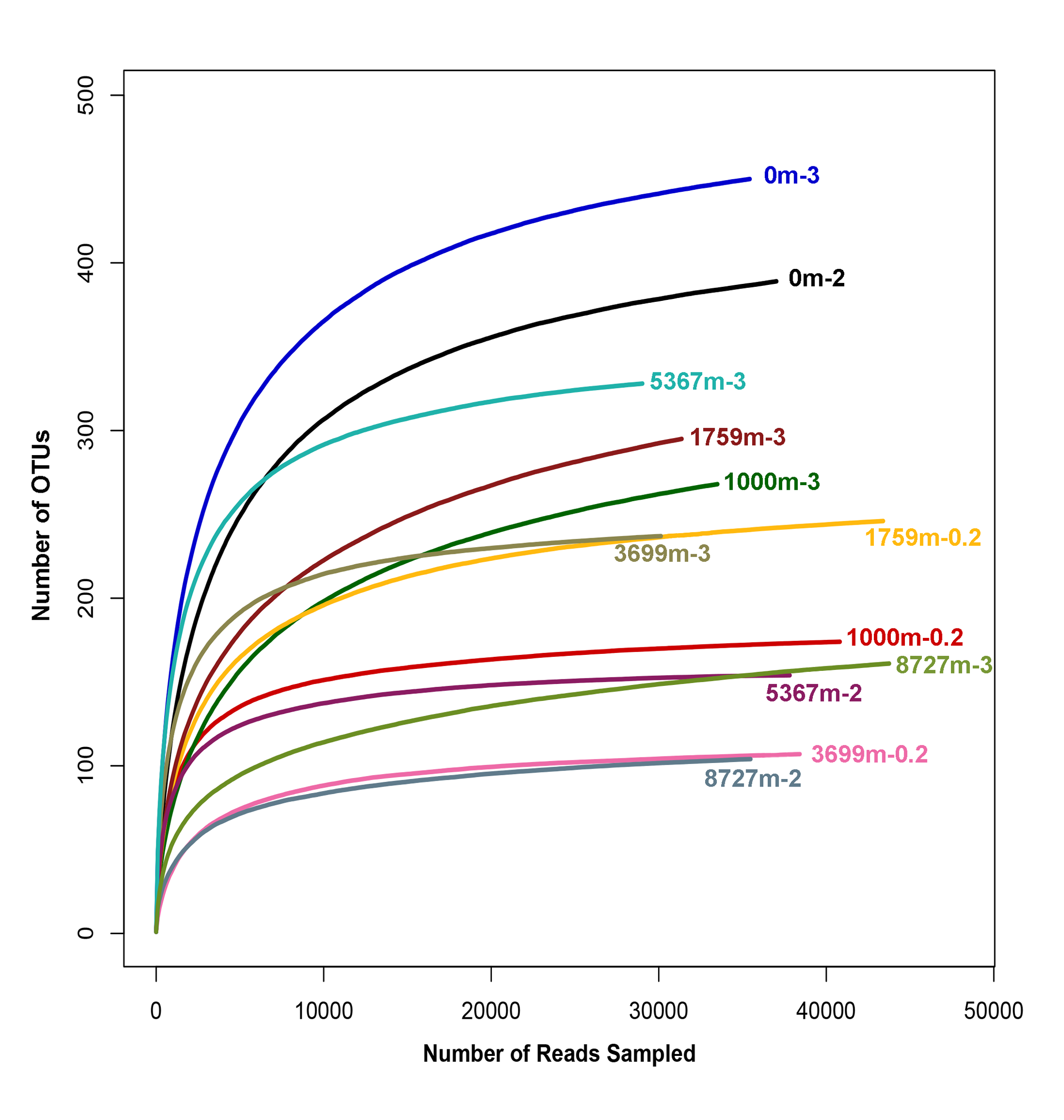

Supplement: FIGURE S3 — Rarefaction curves for samples in this study. Left half of a sample name refers to depth and right half refers to size fraction (0.2 refers to 0.2–3 μm while 3 refers to >3 μm). The curve near to saturation indicates that the sequencing effort has exhaustively sampled the diversity. Metazoan sequences are included here. [file Image_3.TIF]

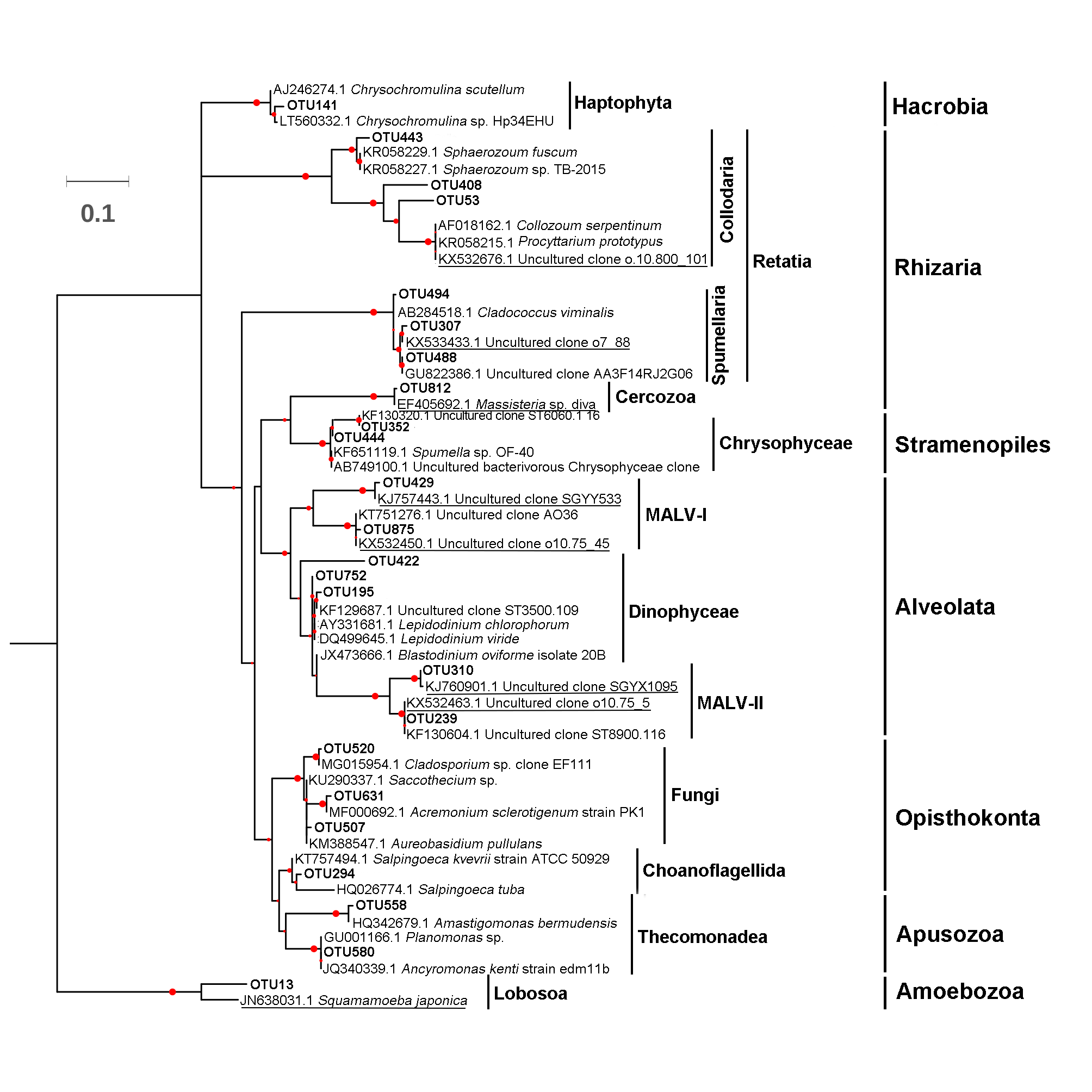

Supplement: FIGURE S4 — Maximum likelihood phylogenetic tree of abundant OTUs in this study. Names in bold correspond to abundant OTUs found in this study while other names (in regular) refer to the nearest cultivated species or uncultured clones with accession numbers on the left (related deep-sea species were underlined). Bootstrap values above 80% are indicated by red solid circles (larger sizes represent higher values) near the nodes. Their taxonomic affiliations were shown on the right at the super group and lower group level (more specific for Rhizaria). [file Image_4.TIF]
